# Phage-encoded depolymerases as a strategy for combating multidrug-resistant *Acinetobacter baumannii*

**DOI:** 10.3389/fcimb.2024.1462620

**Published:** 2024-10-24

**Authors:** Md Minarul Islam, Nasir Uddin Mahbub, Woo Shik Shin, Man Hwan Oh

**Affiliations:** ^1^ Department of Microbiology, College of Science and Technology, Dankook University, Cheonan, Republic of Korea; ^2^ Smart Animal Bio Institute, Dankook University, Cheonan, Republic of Korea; ^3^ Department of Biomedical Sciences and Institute for Medical Science, Jeonbuk National University Medical School, Jeonju, Republic of Korea; ^4^ Department of Pharmaceutical Sciences, Northeast Ohio Medical University, Rootstown, OH, United States; ^5^ Center for Bio-Medical Engineering Core Facility, Dankook University, Cheonan, Republic of Korea

**Keywords:** bacteriophage, depolymerase, polysaccharide, biofilm, drug resistance

## Abstract

*Acinetobacter baumannii*, a predominant nosocomial pathogen, represents a grave threat to public health due to its multiple antimicrobial resistance. Managing patients afflicted with severe infections caused by multiple drug-resistant *A. baumannii* is particularly challenging, given the associated high mortality rates and unfavorable prognoses. The diminishing efficacy of antibiotics against this superbug underscores the urgent necessity for novel treatments or strategies to address this formidable issue. Bacteriophage-derived polysaccharide depolymerase enzymes present a potential approach to combating this pathogen. These enzymes target and degrade the bacterial cell’s exopolysaccharide, capsular polysaccharide, and lipopolysaccharide, thereby disrupting biofilm formation and impairing the bacteria’s defense mechanisms. Nonetheless, the narrow host range of phage depolymerases limits their therapeutic efficacy. Despite the benefits of these enzymes, phage-resistant strains have been identified, highlighting the complexity of phage-host interactions and the need for further investigation. While preliminary findings are encouraging, current investigations are limited, and clinical trials are imperative to advance this treatment approach for broader clinical applications. This review explores the potential of phage-derived depolymerase enzymes against *A. baumannii* infections.

## 1 Introduction

*Acinetobacter baumannii (A. baumannii)* has emerged as one of the most formidable multidrug-resistant (MDR) nosocomial pathogens. The primary complications associated with infections by various MDR *A. baumannii* strains include pneumonia, bloodstream infections, urinary tract infections, skin and wound infections (Nguyen and Joshi, 2021). The World Health Organization (WHO, 2024) and the Centers for Disease Control and Prevention (CDC, 2019) have designated this bacterium as a critical priority pathogen due to the scarcity of effective treatment options, emphasizing the urgent need for additional research to address this challenge.

The exopolysaccharide (EPS), capsular polysaccharide (CPS), lipo-oligosaccharide (LOS), outer membrane protein, pili, adhesion, metal ion uptake system etc. of *A. baumannii* have been identified as the major virulence factors based on numerous studies (Geisinger and Isberg, 2015; Talyansky et al., 2021; Zhao et al., 2021; Karampatakis et al., 2024). Studies involving mutant strains lacking capsular functionality have elucidated their roles in proliferating within soft tissue infection sites, inducing lethality in murine septicemia models, providing defense against serum-mediated killing, and modulating biofilm formation (Russo et al., 2010; Lees-Miller et al., 2013). EPSs play a pivotal role in facilitating bacterial aggregation, leading to the formation of multicellular consortia, wherein the biofilm operates as a highly protected multicellular system. The dense EPS matrix constitutes an effective barrier that impedes the penetration of antibiotics to different layers of the biofilm (Singh et al., 2021). Pathogenic bacteria, including *Staphylococcus aureus*, *A. baumannii*, *Escherichia coli*, *Klebsiella pneumoniae*, *Pseudomonas aeruginosa*, and *Enterococcus faecalis*, have the capacity to form biofilms on medical instrument surfaces, within hospital environments, and on human and animal tissues (Mariani and Galvan, 2023; Weber et al., 2023). Approximately 80% of chronic and recurrent microbial infections in the human body are attributable to bacterial biofilms, which demonstrate antibiotic resistance levels that are 10 to 1000 times higher than those of planktonic cells, thereby substantially contributing to these types of infections (Sharma et al., 2019). In planktonic cells, *A. baumannii* exhibit higher susceptibility to antibiotics (Wences et al., 2022). However, after the establishment of biofilms on appropriate surfaces, they manifest resistance to antibiotics (Al-Shamiri et al., 2021). These biofilms obstruct the ingress of antibiotics into deeper layers, predominantly characterized by polysaccharide constituents, thereby curtailing antibiotic access to bacterial cells (Singh et al., 2021). Hence, there exists a critical imperative to investigate and innovate novel strategies to address the complexities associated with MDR strains of *A. baumannii*.

Bacteriophages have resurfaced as an alternative approach for combating drug-resistant *A. baumannii*, offering a promising avenue for mitigating the challenges posed by antibiotic resistance. Bacteriophages, commonly referred to as phages, are obligate parasitic entities that infects and replicates through either the lytic cycle or integrates its DNA into the host genome in the lysogenic cycle, leading to the production of new phages. These innate bactericidal agents are being revisited to counter bacteria that have developed resistance to multiple antimicrobial agents (Wegrzyn, 2022). Notwithstanding its recognized potential as a substitute for conventional antibiotics, phage therapy encounters efficacy impediments due to the necessity for tailored strategies and the formulation of comprehensive cocktails to address bacterial resistance. The restricted host spectrum and the emergence of phage-resistant bacterial variants are pivotal factors contributing to these challenges (Oechslin, 2018; Pirnay and Kutter, 2021). The coevolution of bacteria and phages drives their selection and infectivity (Wright et al., 2016). Bacteria develop resistance through several mechanisms such as blocking phage receptors, producing extracellular matrices, using CRISPR-Cas and restriction systems to cleave phage DNA, or employing abortive infection systems (Ambroa et al., 2022). Phages, in turn, evolve mutations to counter bacterial defenses. In biofilms, bacteria are shielded from harsh conditions and phage attacks, but phages can evolve enzymes like depolymerases to break down biofilm structures, resulting in a constant arms race between phages and bacteria as they adapt to each other’s defenses (Pires et al., 2021).

A prevalent resistance mechanism observed in *Acinetobacter* entails the secretion of cell-surface capsules, which serve to obscure the primary receptors, rendering it difficult for phages to adhere to the bacterial host (Oromi-Bosch et al., 2023). Nevertheless, researchers have investigated phage-encoded depolymerases capable of enzymatically degrading bacterial EPS, CPS and lipopolysaccharide (LPS) materials. In numerous animal infection models, phage-derived depolymerases are exhibiting therapeutic promise by selectively targeting bacterial capsules and exhibiting antibiotic efficacy (Lin et al., 2014; Chen et al., 2018, 2020). In phage therapy, the efficacy of phages may be compromised due to the frequent development of bacterial resistance against them. Conversely, free depolymerases, distinct from intact phage particles, do not often engender the emergence of resistant bacterial strains. This phenomenon stems from the fact that depolymerases do not directly induce bacterial cell death; instead, they augment bacterial susceptibility to immune responses (Chen et al., 2022; Guo et al., 2023). Hence, phage-derived depolymerases have emerged as innovative alternative antimicrobials targeting MDR bacteria, particularly those harboring polysaccharide capsules that confer resistance to antibiotics. Numerous phages are equipped with tail-associated depolymerases capable of degrading bacterial CPS, EPS, and LPS, thereby uncovering binding sites for antimicrobial agents. Additionally, they facilitate phage attachment, enhance innate immune responses, and optimize antibiotic effectiveness, including penetration into biofilms (Hsieh et al., 2017; Wang et al., 2024a). The aims of this review are to summarize the state-of-the-art knowledge on the phage derived depolymerase enzymes that can degrade polysaccharide materials and their potential role in treating MDR *A. baumannii* infections.

## 2 Envelope associated polysaccharides of *A. baumannii* and their impact on pathogenicity

The capsule that surrounds the *A. baumannii* surface is a crucial fitness and virulence factor. Capsules facilitate bacteria to adhere to biotic and abiotic surfaces promoting colonization in diverse niches and thereby biofilm formation (Kaur and Dey, 2023). Poly-N-acetyl glucosamine (PNAG), an important polysaccharide, is well described as a building block of *A. baumannii* biofilm. In *A. baumannii*, the pgaABCD locus is found to be associated with the production of PNAG. Deletion of the pgaABCD locus led to a reduced biofilm phenotype, which was restored by complementation, demonstrating the role of polysaccharides in biofilm formation (Choi et al., 2009). Composed of repeating polysaccharide units known as K units, this capsule forms a protective coating around the bacterial cell wall, shielding it from environmental stresses such as desiccation and disinfection (Tipton Kyle et al., 2018). The ability of *A. baumannii* to survive in the hospital environment is favored by resistance to disinfectants and desiccation. In *A. baumannii*, polysaccharide materials provide a physical barrier that enhances water retention, allowing the bacteria to survive for long periods of desiccation (Kaur and Dey, 2023). A study showed that the *A. baumannii* strain AB5075 wild-type virulent opaque cells produced a capsule with a 2-fold increased thickness than avirulent translucent cells. In desiccation and disinfectants, virulent opaque cells showed higher survival on dry surfaces compared to avirulent translucent cells (Chin et al., 2018). Besides, CPS also involves other virulence factors such as motility, confirming its role as a determinant of virulence (Rakovitsky et al., 2021).

Additionally, the *A. baumannii* capsule plays a vital role in defending against host cell killing and evading activation of the innate immune response (Akoolo et al., 2022). A capsular mutant, designated as wza, of *A. baumannii* exhibited heightened susceptibility to whole blood, complement, and neutrophil-mediated killing, underscoring the critical role of the polysaccharide capsule in immune evasion (Bjanes et al., 2023). The capsule is also known to play a role in phagocytosis. In hyper mucoid *A. baumannii*, the capsule assembly gene gtr6 protects it from phagocytosis by inhibiting the deposition of complement component 3 (Talyansky et al., 2021; Gong et al., 2022). In a study, wild-type mice with Toll-like receptor 4 died from *A. baumannii*-induced septic shock, while TLR4-deficient mice survived despite having a similar bacterial load. The hypervirulent *A. baumannii* releases more LPS, which activates TLR4, leading to lethal sepsis in the host. The LpxC gene, involved in lipid A biosynthesis, (a component of LPS), is targeted by LpxC inhibitor. Though the inhibitor did not inhibit bacterial growth, it suppressed TLR4 activation by *A. baumannii*. Mice treated with the LpxC inhibitor showed enhanced opsonophagocytic killing, reduced serum LPS concentrations and inflammation, and were completely protected from lethal infection (Lin et al., 2012). The anti-OmpA monoclonal antibody enhanced macrophages to kill the K1 capsule-negative mutant *A. baumannii 307.30*, except for those containing thick capsules, particularly extensively drug-resistant *A. baumannii*. The binding affinity of monoclonal antibodies with drug-resistant clinical isolates of *A. baumannii* showed weaker affinity than that between mABs and ATCC19606, possibly due to capsular polysaccharide blocking MAb access to OmpA (Wang-Lin Shun et al., 2017).

In addition to protect from host defense system, capsules also confer resistance to a wide range of antibiotics by limiting the penetration of antibiotics to the outer membrane where the antibiotics can access the inner membrane. Geisinger and Isberg (Geisinger and Isberg, 2015) reported that when *A. baumannii* is exposed to sub inhibitory concentrations of chloramphenicol or erythromycin, it leads to increased production of capsular polysaccharide. This elevated CPS production is reversible and non-mutational, and occurs alongside with heightened resistance to the antibiotic independent of the K locus. Colony phase variation by *A. baumannii* responds to differential alterations in tolerance to antibiotics. Mushtaq et al. (Mushtaq et al., 2024) demonstrated that in opaque variants of *A. baumannii AB5075*, extracellular polysaccharide material plays a role in colistin tolerance at the single-cell level. At the community level, the opaque variant forms a mushroom-shaped biofilm. Thus, demonstrating its fitness advantage over the translucent variants and its ability to tolerate colistin.

## 3 Bacteriophage-encoded enzymes with depolymerizing activity against bacterial polysaccharides

**Figure 1:**
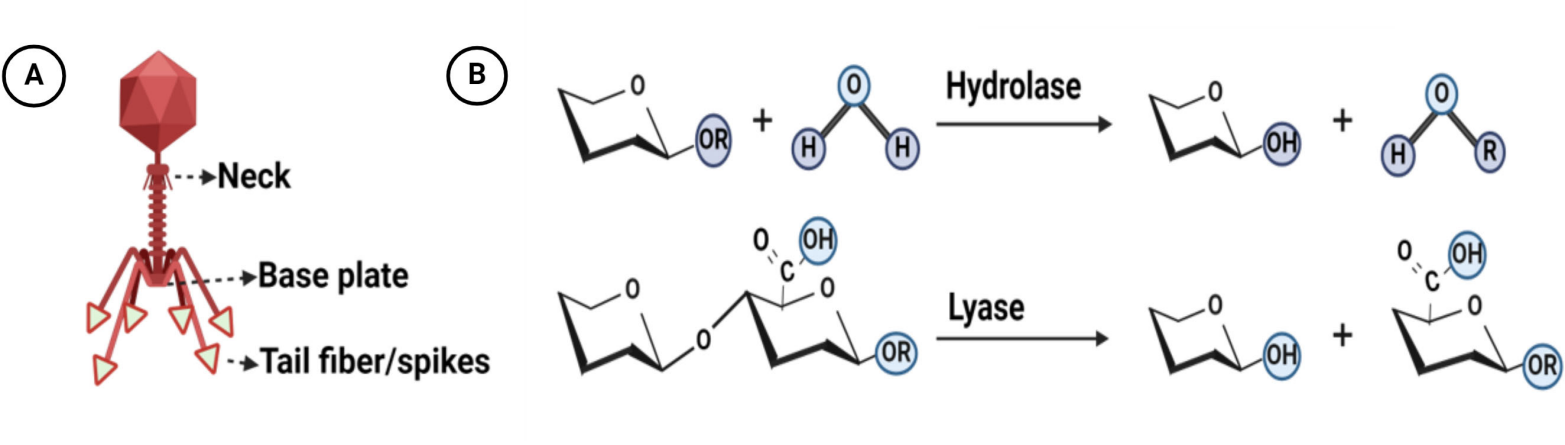
Structure and mechanisms of action of bacteriophage depolymerases: **(A)** Distribution of depolymerase within the structure of bacteriophages **(B)** The overarching mechanisms by which depolymerases catalyze polysaccharide degradation involve two fundamental processes: hydrolysis and lysis. During hydrolysis, depolymerases enzymatically cleave the glycosidic bonds between sugar monomers, resulting in the fragmentation of polysaccharides into smaller sugar units. In the lysis process, these enzymes compromise the structural integrity of the polysaccharide matrix. Created with BioRender.com.

**Figure 2:**
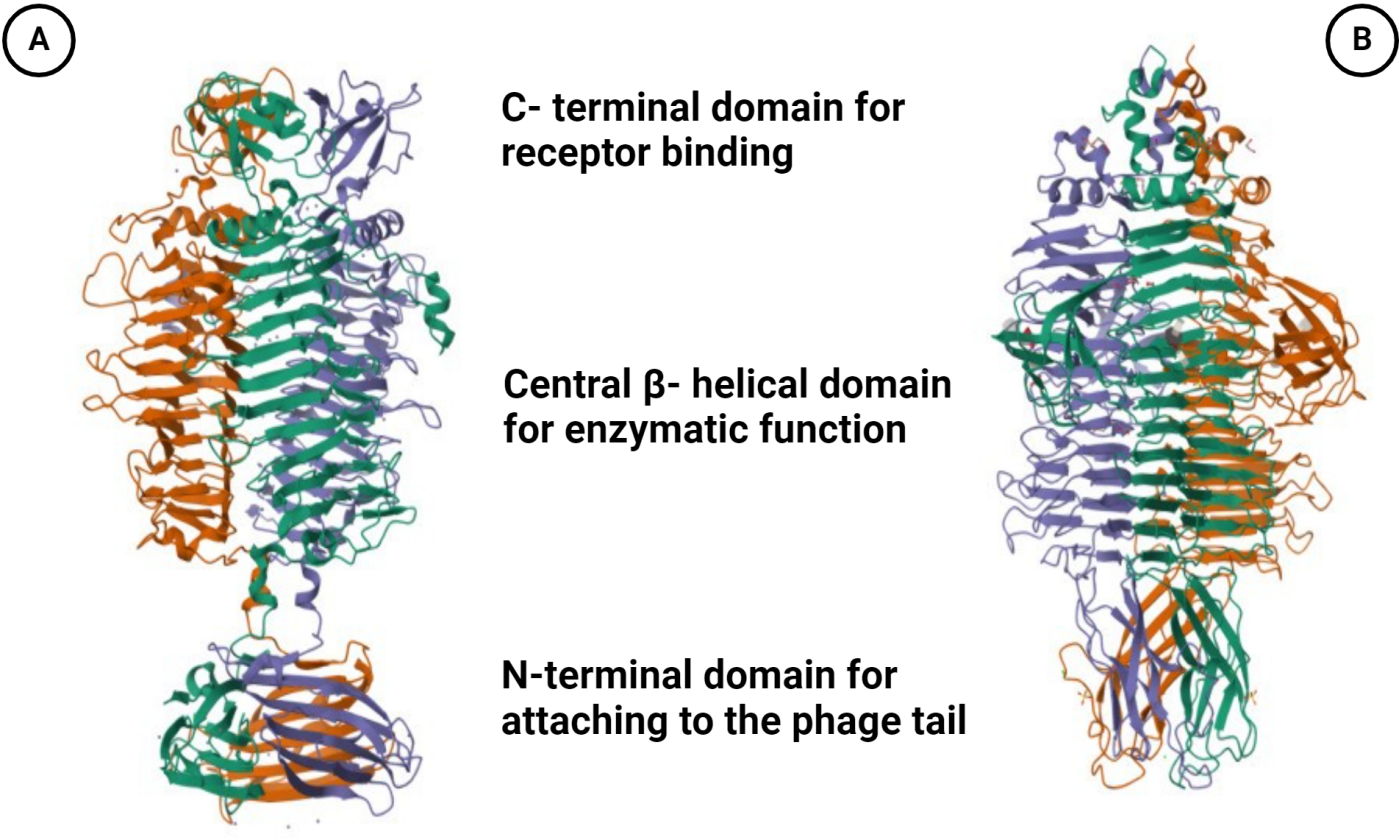
Illustration is the trimeric crystal structure of the tail spike protein. **(A)** gp49 tail spike protein (PDB ID: 6C72) derived from *A. baumannii* bacteriophage Fri1, a capsular polysaccharide depolymerase (Hydrolase). **(B)** Gp54 tail spike (PDB ID: 4Y9V) of *A baumannii* bacteriophage AP22 polysaccharide degrading lyase. Three monomers are marked by different colors. Created with BioRender.com.

Phage depolymerases represent enzymatic proteins designed to dismantle polymers, particularly targeting bacterial outer membrane polysaccharides essential for bacteriophage adsorption. These enzymatic entities play a crucial role in facilitating phage attachment to the bacterial cell surface and subsequent degradation of the bacterial capsule (Knecht et al., 2019). Tail fibers or tail spikes, integral components of bacteriophage structure, serve as anchoring points for depolymerases, which are linked to the base plate of the phage, with a few exceptions found on the neck (Lai et al., 2016; Pires et al., 2016; Blundell-Hunter et al., 2021) (**Figure 1A**). Biochemically, depolymerases are dichotomized into two distinct types: lyases and hydrolases (Cai et al., 2023; Magill and Skvortsov, 2023). Hydrolases break glycosidic bonds by reacting with water, disrupting the glycosyl-oxygen bond. Lyases, on the other hand, cleave glycosidic bonds through β-elimination, creating a new double bond without the need for water (Li et al., 2022) (**Figure 1B**). Predominantly harbored in receptor binding proteins (RBPs), depolymerases are crucial for triggering phage infection through the targeting and enzymatic disassembly of bacterial capsules at the onset (Latka et al., 2019). Receptor binding proteins (RBPs) possessing depolymerase activity exhibit a modular architecture comprising three distinct domains: An N-terminal domain responsible for tethering to the phage tail, a central β-helical domain facilitating enzymatic function, and a C-terminal domain associated with receptor binding and potential chaperone activity (Li et al., 2022; Guo et al., 2023; Klumpp et al., 2023; Maciejewska et al., 2023) (**Figure 2**). The modular configuration of RBPs enables expedited alterations in host specificity, accomplished via horizontal gene transfer such as transduction, which mediates the interchange of host-specific central domains, and vertical gene transfer, a process marked by the accrual of mutations within polysaccharide-depolymerizing domains (Matilla and Salmond, 2014; Latka et al., 2017, 2019; Pas et al., 2023). Two variants of phage depolymerases are discernible: one bound within phage particles and the other existing as soluble proteins following host cell lysis (Drulis-Kawa et al., 2015; Wang et al., 2019). Ultimately, the structural diversity and modular nature of phage depolymerases are fundamental to their ability to degrade bacterial polysaccharides, thereby promoting efficient phage-host interactions and successful infection.

## 4 Bacteriophage-mediated depolymerases combating *A. baumannii*

**Figure 3:**
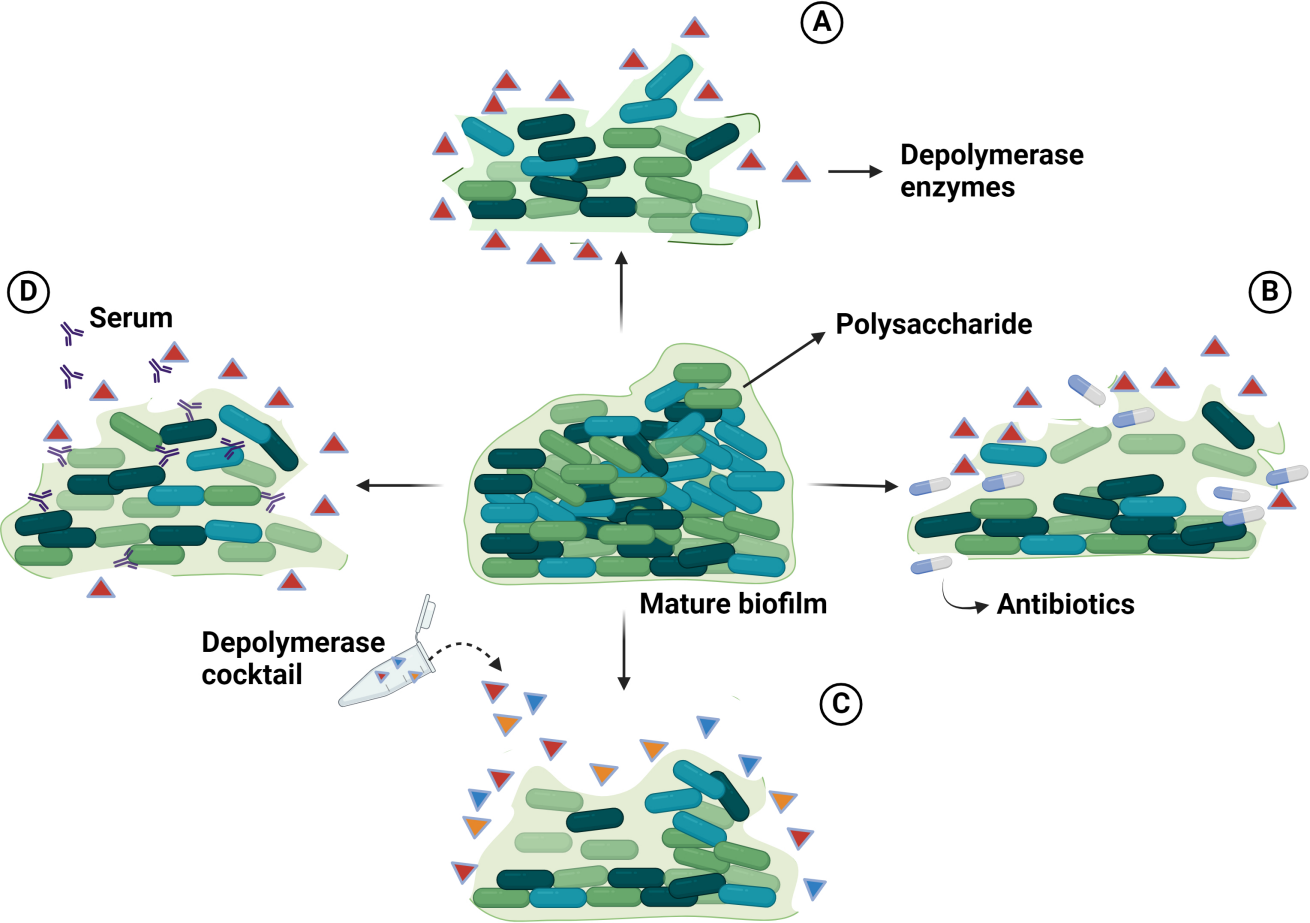
The illustration delineates the anti-biofilm mechanisms of phage-encoded depolymerases: **(A)** Phage-encoded depolymerases impede biofilm formation by degrading the extracellular polysaccharides that protect the bacterial cells within the biofilm. **(B)** The synergistic application of phage-encoded depolymerases and antibiotics enhances the bactericidal efficacy against *A. baumannii* biofilms. The depolymerases degrade the extracellular polysaccharide, rendering the bacteria more susceptible to antibiotic treatment. **(C)** A consortium of phage-encoded depolymerases can effectively dismantle biofilms by degrading the polysaccharide matrix. **(D)** Phage-encoded depolymerases sensitize bacteria to the host immune response by degrading the protective polysaccharide matrix of biofilms. Created with BioRender.com.

Capsular polysaccharide and biofilm of *A. baumannii* are virulence determinants that play a major role in resistance to antimicrobial agents, evasion of host immune responses and adaptation to other stresses (Geisinger and Isberg, 2015; Mendes et al., 2023). These structural components act as protective barriers, hindering the access of various antimicrobial agents to their respective binding sites (Roy et al., 2022). Biofilms are complex assemblies of bacteria adhering to surfaces and enclosed within a matrix composed of extracellular polymeric substances, comprising proteins, lipids, nucleic acids, polysaccharides, and other constituents. In response to the challenge of biofilm resistance, alternative approaches such as the utilization of phage depolymerases have been explored. Encouragingly, phages carrying depolymerases possess the capacity to degrade the polysaccharide constituents of both capsules and biofilms (**Figure 3**). The polysaccharide components present on the outer surface of *A. baumannii* cells are the primary targets for bacteriophages harboring specific structural depolymerases. These depolymerases are crucial for mediating the initial interaction between the phage and the host bacterium (Drobiazko et al., 2022). A solitary phage depolymerase possesses the capacity to address bacterial surface polysaccharides and biofilms. EPSs are crucial for the attachment of bacterial cells together and forming multicellular consortia in biofilms. They are vital for the structural stability, functionality, and virulence of bacterial biofilms (Karygianni et al., 2020). The dense EPS matrix has a high binding affinity to antimicrobial agents, creating a barrier that hinders antibiotic diffusion into biofilm layers (Sharma et al., 2023). Phage-encoded depolymerase enzymes target EPS by recognizing, binding to, and degrading the polysaccharide matrix of bacterial cell walls. This enzymatic action disrupts biofilm structure, reducing bacterial virulence and increasing susceptibility to the host immune system (Tian et al., 2021). Depolymerases degrade polysaccharides like capsular CPSs, LOSs, O-polysaccharides, and EPSs in biofilms. Hydrolase break down α-1,4-glycosidic bonds in polysaccharides to produce oligosaccharides through hydrolysis (Wang et al., 2024b). Lyases, on the other hand, cleave anionic polyuronides by a β-elimination reaction. This process involves removing a proton from the C5 position of the uronide ring and breaking the ether linkage at the C4 position with the help of a general acid-base catalyst, resulting in an unsaturated product (Pandey et al., 2023).

However, achieving comprehensive eradication of these virulence determinants may necessitate the utilization of combinations of phage depolymerase cocktails or their concurrent administration with antibiotics, or they may serve as adjuncts. Hence, the utilization of phage depolymerases emerges as a prospective tactic for addressing *A. baumannii* infections, offering potential as standalone interventions or in tandem with antibiotic therapies. This underscores the promising trajectory of phage-derived depolymerases in the realm of antimicrobial strategies against this resilient pathogen.

### 4.1 Employing a singular phage-derived depolymerase for mitigating the virulence of pathogenic *A. baumannii*

A single phage depolymerase can efficiently target and disperse the biofilm matrix of *Acinetobacter*. By degrading the extracellular polymeric substances that shield and reinforce the biofilm, this enzyme disrupts its structure, rendering the bacterial cells more susceptible to treatment (**Figure 3A**). This enzymatic action aids in facilitating the phage’s DNA injection into the bacterial host. Unlike certain other phage-encoded enzymes, depolymerases do not induce direct bacterial cell lysis. Rather, they degrade bacterial CPS, rendering them vulnerable to host immune responses and antibacterial treatments (Dicks and Vermeulen, 2024). Hydrolases are enzymatic catalysts that cleave glycosidic bonds by disrupting the glycosyl-oxygen bond. Within this category, various subclasses exist, including sialidases, levanases, xylosidases, dextranases and rhamnosidases. Conversely, lyases function by catalyzing the separation of the glycosidic link between monosaccharides and the C4 position in uronic acid molecules. This process involves the creation of a double bond via the β-elimination mechanism between the C4 and C5 positions in nonreducing uronic acid molecules. Examples of lyases include hyaluronidases, alginate lyases, and pectin/pectate lyases (Cai et al., 2023). The utilization of depolymerases for antibacterial purposes can occur through two approaches: as enzymes expressed by bacteriophages and as recombinant protein; and both strategies can be used in combination with antibiotics to broaden their effectiveness against a wider range of hosts (Wang et al., 2024a). These bacteriophages possess inherent mechanisms for recognizing specific bacterial strains, facilitating targeted interventions (Hibstu et al., 2022). Moreover, their capacity to deliver depolymerases directly to infection sites and penetrate biofilms presents advantages over conventional antibiotics (Jo et al., 2023). Recombinant protein synthesis enables standardized, large-scale production of depolymerases with stringent quality control measures, ensuring uniform potency and reliability. Furthermore, genetic engineering methodologies offer avenues for refining recombinant depolymerases to augment their stability, enzymatic activity, or specificity towards various polysaccharide targets (Drulis-Kawa et al., 2015; Hassan et al., 2021; Noreika et al., 2023).

PghP is a bacteriophage-encoded peptidase that specifically hydrolyzes γ-polyglutamic acid (γ-PGA), a polymeric extracellular barrier produced by certain bacterial species. This enzymatic activity is critical for bacteriophage entry, as PghP degrades the γ-PGA matrix surrounding the bacteria. PghP catalyzes the cleavage of γ-PGA into smaller oligo-γ-glutamates, further processing these into tri-, tetra-, and penta-γ-glutamate units (Kimura and Itoh, 2003). Sialidases, also referred to as neuraminidases, are enzymes that catalyze the cleavage of the α-linkage of terminal sialic acid residues in glycoconjugates (Keil et al., 2022). Pathogenic bacteria, including *Escherichia coli K1, Acinetobacter baumannii, Haemophilus influenzae*, and *Neisseria* spp., exploit sialic acid to evade host immune defenses by incorporating it into their surface structures or utilizing it as a metabolic nutrient source (Ko et al., 2018; Jennings et al., 2022). Furthermore, numerous bacterial species produce sialidases to enhance their survival within mucosal environments and facilitate interactions with other microorganisms, thereby contributing to their pathogenic potential (Lewis and Lewis, 2012; Tailford et al., 2015). Bacteriophages encode endosialidases that degrade capsular polysaccharides, rendering bacteria more vulnerable to host immune responses. Studies have demonstrated that phage-derived endosialidases can sensitize *Escherichia coli* to immune defenses, promoting phagocytosis and offering protection against systemic infection in murine models (Mushtaq et al., 2004). Levanases (EC 3.2.1.65) are enzymes present in microorganisms such as *Bacillus* and *Pseudomonas*, which catalyze the hydrolysis of the β-2,6-linked chains of levan, a fructan polymer (Murakami et al., 1992). Bacteriophage ϕNIT1 possesses the pghP gene, encoding a γ-PGA hydrolase that enables the phage to overcome the host cell’s defense mechanisms. Additionally, ϕNIT1 contains a gene encoding LevP, an active levan hydrolase classified as an endo-levanase, which cleaves levan, an exopolysaccharide produced by *Bacillus subtilis* (Ozaki et al., 2017). Xylosidases (EC 3.2.1.37), dextranases (EC 3.2.1.11), lipases (EC 3.1.1.3), and rhamnosidases (EC 3.2.1.40) represent enzyme classes that are among the least frequently identified depolymerase domains in bacteriophages. These enzymes are involved in the hydrolytic degradation of specific substrates: xylan, dextran, triacylglycerols, and rhamnogalacturonan, respectively. Hyaluronate lyases (EC 4.2.99.1) and hyaluronidases (EC 4.2.2.1) are enzymes responsible for the degradation of hyaluronate. Phage-encoded hyaluronidases, such as those identified in *Streptococcus pyogenes* bacteriophages, specifically target hyaluronan, aiding in the degradation of the bacterial hyaluronan capsule (Baker et al., 2002). Alginate lyases (EC 4.2.2) are enzymes that catalyze the degradation of alginic acids, found in organisms such as *Pseudomonas aeruginosa* and *Azotobacter vinelandii* phages. These enzymes, including mannuronate lyase (EC 4.2.2.3) and guluronate lyase (EC 4.2.2.11), specifically target alginates, which serve as structural components in brown algae and bacterial extracellular polysaccharides (EPS) (Kim et al., 2011).

Pectin, a polysaccharide with an α-1,4-linked D-galacturonic acid backbone and α-1,2-l-rhamnose units, is catalyzed by pectin/pectate lyase, which cleaves the α-1,4 bonds of polygalacturonic acid is catalyzed by pectate lyase, which cleaves the α-1,4 bonds of polygalacturonic acid (Latka et al., 2017; Minzanova et al., 2018), are typically present in the cell wall or capsule of *A. baumannii.* DpoMK34, a pectate lyase, derived from the *A. baumannii* MK34 phage, demonstrates considerable efficacy even at low concentrations, falling within sub-micromolar ranges. Its activity is not limited to *A. baumannii* MK34 but also extends to strain CIP110467, which possesses a K2 capsular serotype, suggesting its potential for broad-spectrum effectiveness (Abdelkader et al., 2022). A study revealed the presence of 12 podoviruses featuring depolymerases equipped with a pectate lyase domain, facilitating the breakdown of capsules within the *Acinetobacter baumannii-Acinetobacter calcoaceticus* complex. This observation implies that these *podoviruses* may have undergone evolutionary refinement through the acquisition of specialized pectate lyase coding regions, thereby enhancing their infectivity and viability within this bacterial cohort (Oliveira et al., 2017). Dpo48, recognized as a capsule depolymerase, displays a broad activity spectrum, and maintains effectiveness across varied pH and temperature conditions. Its capacity to efficiently strip capsules from *A. baumannii*, even at elevated concentrations, facilitates bacterial eradication by serum complement *in vitro* (Liu et al., 2019). The *Acinetobacter baumannii* phage Bϕ-R2096, sourced from sewage water, exhibits robust bacteriolytic activity and generates distinct circular plaques surrounded by halos, indicative of the potential presence of phage-derived depolymerases. *In vivo* investigations utilizing *Galleria mellonella* larvae and mice infected with carbapenem-resistant *A. baumannii* (CRAB) reveal that Bϕ-R2096 significantly enhances survival rates and mitigates histologic lung damage in infected hosts, with no reported mortality or adverse effects (Jeon et al., 2019).

A study by Hernandez-Morales et al. (Hernandez-Morales et al., 2018) investigated the bacteriophage Petty, which harbors the depolymerase gene Dpo1, capable of degrading exopolysaccharides and infecting both *A. nosocomialis* and *A. baumannii*. *In vitro* assessments revealed that Dpo1 effectively reduced the viscosity of EPS and partially disrupted biofilms formed by the tested *Acinetobacter* strains, resulting in a modest reduction of approximately 20%. Although complete eradication of the biofilms was not achieved, Dpo1 likely attenuated the virulence of the examined strains by degrading EPS components. Notably, plaques formed by Petty exhibited halos, indicative of depolymerase activity targeting capsular exopolysaccharides. Additionally, gene 39, encoding a putative tail fiber, displayed depolymerase activity, suggesting its potential application in diagnostics and therapeutics against drug-resistant *Acinetobacter* strains through biofilm disruption. Another investigation revealed that depolymerases sourced from bacteriophages AM24, BS46, and APK09 possess the capability to degrade the K9 CPS of the antibiotic-resistant *A. baumannii* GC1(ST1IP) isolate SGH0807. Upon exposure to the K70 CPS from SGH0807, these depolymerases induced the cleavage of monomers and dimers at the K-unit linkage, resulting in the generation of oligosaccharide fragments. This finding underscores the potential of these depolymerases as a targeted strategy against the antibiotic-resistant strain by degrading its K70 CPS. Moreover, the observed specificity of these phages’ depolymerases for distinct CPS types suggests their potential efficacy in infecting and eradicating the SGH0807 K70 isolate (Kasimova et al., 2023).

Timoshina et al. (Timoshina et al., 2023) made an intriguing discovery of seven novel *Friunaviruses* and proceeded to dissect their functionalities and mechanisms in the degradation of CPS. Recombinant depolymerases were expressed in *Escherichia coli B834* (APK09_gp48, APK14_gp49, APK16_gp47, APK86_gp49, APK127v_gp47, APK128_gp45, and APK37.1_gp49) generated, demonstrating effective degradation of the CPS of *A. baumannii* under laboratory conditions. Remarkably, recombinant depolymerase APK09_gp48 exhibited significant efficacy in reducing mortality rates among *Galleria mellonella* larvae infected with *A. baumannii* of K9 capsular type. Moreover, when administered independently, TSD APK09_gp48 did not result in larval mortality, underscoring its safety. These outcomes highlight the potential therapeutic utility of depolymerase APK09_gp48 in combating *A. baumannii* infections. Eight bacteriophages were identified, each carrying depolymerase genes. These genes were cloned, expressed, and the resulting enzymes were purified. The enzymes broke down the capsular polysaccharides (CPSs) of their *A. baumannii* hosts into monomers or dimers of CPS repeats (K units) through a hydrolytic process. Specific depolymerases, such as APK2_gp43, APK32_gp46, APK37_gp44, APK44_gp44, APK48_gp43, APK87_gp48, APK89_gp46, and APK116_gp43, were found to be glycosidases. Notably, APK2_gp43 could interact with both K2 and K93 CPSs because of their similar K unit structures and identical linkages, enabling the enzyme to break down both types effectively (Popova et al., 2021). The recombinant depolymerase derived from the tail spike protein (TSP) of the bacteriophage φAB6 exhibits proficient degradation of *A. baumannii* biofilms *in vitro*. It effectively impedes biofilm formation and dismantles pre-existing biofilms. Even at minimal concentrations, TSP significantly hampers biofilm formation and adhesion on Foley catheter surfaces. Furthermore, it induces bacterial cell death and augments survival rates in zebrafish, suggesting its potential as a novel antibiotic against MDR *A. baumannii* and as a biocontrol agent for preventing biofilm formation on medical devices (Shahed-Al-Mahmud et al., 2021).

Depolymerases used in therapeutic applications are typically prepared as liquid formulations for parenteral injections, aerosols for inhalations and topical delivery methods also in use (Maciejewska et al., 2018). The enzyme production requires a comprehensive protein purification process to remove bacterial endotoxins. The delivery of enzymes through aerosolization and topical application allows for targeted drug accumulation at the infection site while minimizing systemic exposure (Ryan et al., 2011). Specific pH and buffers are required for long-term storage or to ensure delivery to the reaction site. Chen et al. (Chen et al., 2022) found that storing Dpo71 in phosphate-buffered saline at 4°C maintained its stability for up to six months without significant loss of activity. Another study showed that KP32gp37 enzyme was resistant to SDS detergent and trypsin protease, while KP32gp38 was not (Majkowska-Skrobek et al., 2018). These findings provide safe formulation strategies for depolymerases for therapeutic use to prevent denaturation by body fluids.

In general, the mechanism of action for phage depolymerases involves the cleavage of glycosidic bonds present within bacterial polysaccharides, leading to the formation of oligosaccharide products either through hydrolysis or lysis. The EPS found in *A. baumannii* biofilms exhibit three distinct bond types: β-1,6-glycosidic, β-1,3-glycosidic and β-2,6-glycosidic bond (Lee et al., 2017). Each phage depolymerase possesses the ability to hydrolyze at least one of these bond types (Guo et al., 2023). Hence, employing a combination of phage depolymerases in a cocktail may present a promising approach for the comprehensive eradication of *A. baumannii* biofilms.

### 4.2 Combining phage depolymerase with antibiotics has shown synergistic effects against *A. baumannii*

Integrating antibiotics with phage-derived depolymerases offers a potent approach to addressing MDR bacterial infections. Depolymerases alone might be less effective due to biofilm variability; however, their combination with antibiotics increases bacterial susceptibility by breaking down protective polysaccharide matrices. This combined mechanism enhances antibiotic penetration, making the treatment more effective in eradicating infections, particularly those caused by resistant bacteria (Topka-Bielecka et al., 2021) (**Figure 3B**).

Colistin is considered a last-resort antibiotic used to treat infections caused by MDR *A. baumannii*. However, there have been reports of *A. baumannii* strains developing resistance to colistin (Elham and Fawzia, 2019; Seleim et al., 2022). Combining phage depolymerases with colistin could help to address this issue. The synergistic use of colistin and phage depolymerases, such as Dpo71, could effectively counter MDR *A. baumannii*. Dpo71, a phage-derived enzyme, boosts bacterial susceptibility to immune system attacks and enhances colistin’s antimicrobial activity (Chen et al., 2022). It helps to clears bacteria by human serum, degrades biofilms, and improves survival outcomes in infection models. The strain-specific action of Dpo71 demonstrates its potential as a complementary therapy to antibiotics against resistant bacterial infections (Chen et al., 2022). Phage vWU2001, which was isolated from treated wastewater, produced large plaques with halo zones, suggesting the presence of a phage-encoded depolymerase. When combined with colistin, phage vWU2001 significantly inhibited the growth and viability of carbapenem-resistant *A. baumannii* more effectively than either treatment alone. This combination also improved the survival of *G. mellonella* and enhanced bacterial clearance, indicating a synergistic effect (Wintachai et al., 2022). Here, phage depolymerases might act as an adjuvant that could help prevent the emergence of resistant cells through synergistic interactions.

### 4.3 Employing a phage depolymerase cocktails strategy

Single-phage depolymerase therapy exhibits dose-dependent repression of bacterial proliferation, albeit encountering impediments such as the emergence of phage-resistant bacterial strains, particularly a problem in the case of *A. baumannii*. To mitigate this challenge, the utilization of cocktails comprising multiple phages encoding depolymerases exhibiting diverse host specificities could demonstrate heightened effectiveness by broadening the host range and mitigating resistance occurrences (**Figure 3C**). Additionally, the incorporation of multiple depolymerases within these cocktails addresses the inherent limitations in the specificity of individual depolymerases for eradicating bacterial biofilms. In sum, the employment of phage depolymerase cocktails augments therapeutic efficacy by targeting a wider array of bacterial strains, potentially delaying the onset of phage resistance, bolstering antimicrobial effectiveness (Chang et al., 2022; Tan et al., 2022). For instance, the co-administration of Ab105-2phiΔCI404ad with phage vB_AbaP_B3, known for their depolymerase expression, synergistically enhanced the antimicrobial efficacy against diverse strains of *A. baumannii*, encompassing both clinical and reference strains (Blasco et al., 2022).

Phage cocktails are recognized for their ability to enhance antibacterial activity against biofilms. Yet, there is a lack of research focusing on the potential use of various phage depolymerases within these cocktails to target bacterial capsules, especially in the context of *A. baumannii* infections. Bacteriophage cocktails have been tested to treat septicemia (Patel et al., 2021), wound infections (Ilomuanya et al., 2022), nasal infections (Cha et al., 2018), and pneumonia (Li et al., 2024) caused by *A. baumannii* in animal models. In addition, phage cocktails have been effectively used in clinical settings to treat human urinary tract infections (Grygorcewicz et al., 2021), necrotizing pancreatitis (Schooley et al., 2017), and post-operative cerebritis (Lavergne et al., 2018) caused by MDR *A. baumannii*. Further research is needed to investigate the efficacy and safety of phage depolymerase cocktails in treating *A. baumannii* infections.

### 4.4 Phage depolymerases sensitize bacterial pathogens to serum killing for enhanced innate immune activity

Polysaccharide components are crucial for shielding bacteria from immune assaults and adverse environmental conditions, including phage infection. Notably, the application of depolymerases in conjunction with human serum or immune cells has significantly augmented antibacterial efficacy (**Figure 3D**). Phage depolymerases render bacteria more susceptible to human serum, resulting in heightened bacterial eradication. This approach represents a promising strategy for combating infections and surmounting bacterial defenses (Maciejewska et al., 2018). Hugo Oliveira et al. (Oliveira et al., 2019) reported that the recently discovered *myovirus* vB_AbaM_B9, along with its depolymerase B9gp69, effectively dismantles exopolysaccharides sourced from various strains of *A. baumannii*. This functionality increases susceptibility to serum-mediated bacterial elimination while simultaneously reducing the likelihood of bacterial resistance development. Specifically engineered to target the virulent K45 capsule type, this recombinant depolymerase demonstrates potential as an antimicrobial supplement, fortifying the host immune defense against *A. baumannii* infections. The analysis of *A. baumannii* phage IME285 resulted in the detection of a depolymerase gene, ORF49, following genomic scrutiny. Subsequent investigation led to the cloning and expression of the recombinant enzyme, Dp49, originating from this gene, showcasing substantial depolymerase activity both in murine experimental models, and *in vitro*, as evidenced by the formation of translucent halos surrounding bacterial plaques. Dp49 displayed efficacy against 25 of the 49 A*. baumannii* strains tested, amplifying the inhibitory impact of serum on bacterial proliferation *in vitro* and augmenting survival rates in *A. baumannii*-infected mice. These observations indicate the potential utility of Dp49 as a promising intervention for managing infections instigated by MDR *A. baumannii* (Wang et al., 2020). Therefore, there is a good reason to delve into further exploration of depolymerases that can effectively target various bacterial types and their corresponding strains.

## 5 Prospective avenues for phage depolymerase-based interventions

Phage-encoded enzymes, such as polysaccharide depolymerases raised attention as novel antibacterial agents due to their potential to eradicate biofilms and capsular polysaccharides that hinder phage adsorption to host bacteria (Bleriot et al., 2024). In contrast to whole phage particles, bacteria hardly develop resistance against free depolymerases, making them a promising antibacterial agent (Chen et al., 2022). Genetically engineering phage depolymerases to modify host ranges holds considerable promise for phage applications (Anyaegbunam et al., 2022). In Gram-negative *E. coli*, genetically engineered phage enzymes significantly prevent biofilm formation (Lu and Collins, 2007). Following this, engineered depolymerase could be effective in *A. baumannii*. As our comprehension of bacteriophage therapy advances, the engineered phage-depolymerases could become a viable solution for biofilm management across various settings. By synthesizing biofilm-degrading enzymes by genetic engineering during the infection process, these phages can simultaneously target bacterial cells and the biofilm matrix, resulting in more efficient biofilm eradication compared to traditional bacteriophage treatments. This underscores the promise of employing engineered enzymatic bacteriophages in combating bacterial biofilms, showcasing the benefits of synthetic biology in addressing both medical and industrial challenges (Lu and Collins, 2007). Advancements in DNA sequencing and synthetic biology present promising avenues for the enhancement of phage-derived enzymes-based antimicrobials, providing critical solutions to address the antibiotic crisis. Comprehensive analysis of the uncharacterized functional proteins in *A. baumannii* phages, alongside the development of phage-derived enzyme formulations, can significantly improve the efficacy and expand the scope of phage applications.

Despite the promising *in vitro* capabilities of phage depolymerases in polysaccharide degradation, clinical trials assessing their therapeutic effectiveness remain scarce. Further investigation is essential to thoroughly understand the clinical utility of phage depolymerases. This includes the development of robust delivery systems to guarantee the stability of depolymerases during storage and transport and their precise delivery to targeted sites while ensuring safety.

## 6 Conclusion

Phages represent a naturalistic solution in combating MDR strains, offering a novel approach to addressing antibiotic resistance. Depolymerases, derived from phages, present a distinct mechanism for combating antibiotic-resistant pathogens, mitigating the risk of resistant strain development. These phage-derived depolymerases exhibit potent antimicrobial properties as alternative antibiotic, in combination with antibiotic, in phage cocktails and as adjuvants by targeting bacterial capsular polysaccharide, particularly effective against biofilm-related infections. This strategy signifies a significant avenue in the ongoing battle against antibiotic resistance. A number of depolymerases from several studies have been identified as promising alternatives to antibiotics against *A.baumannii*, including TSP from the φAB6 phage, Dp49, and Dpo71. However, further research is warranted to identify and characterize additional depolymerases capable of targeting a diverse range of bacterial species and strains comprehensively. Such endeavors hold the potential to significantly contribute to the development of novel antimicrobial strategies and enhance our ability to combat infectious diseases caused by antibiotic-resistant pathogens.
